# The Impact of Drain and Reinforcement on the Outcomes of Bariatric Surgery: A Prospective Study

**DOI:** 10.7759/cureus.20382

**Published:** 2021-12-13

**Authors:** Mohamed A Salman, Ahmed Safina, Ahmed Salman, Mohamed Farah, Khaled Noureldin, Mohamed Issa, Ahmed Dorra, Mohamed Tourky, Hossam El-Din Shaaban, Mohammed Aradaib

**Affiliations:** 1 General Surgery, KasrAlAiny School of Medicine, Cairo University, Cairo, EGY; 2 General surgery, KasrAlAiny School of Medicine, Cairo University, Cairo, EGY; 3 Internal Medicine, KasrAlAiny School of Medicine, Cairo University, Cairo, EGY; 4 Faculty of Medicine, University of Khartoum, Khartoum, SDN; 5 Urology, Sunderland Royal Hospital, Sunderland, GBR; 6 Colorectal Surgery, Southend University Hospital, NHS Trust, Essex, GBR; 7 Surgery, Wirral University Teaching Hospital, Wirral, GBR; 8 Surgery, Prince Charles Hospital, Myrther Tydfil, GBR; 9 Surgery, Leighton Hospital, Crewe, GBR; 10 Surgery, Great Western Hospital, NHS Foundation Trust, London, GBR; 11 Gastroenterology and Hepatology, National Hepatology and Tropical Medicine Research Institute, Cairo, EGY; 12 General Surgery, Sunderland Royal Hospital, Sunderland, GBR

**Keywords:** surgery, bariatric, complications, reinforcement, drain, bariatric surgery

## Abstract

Purpose

We aimed to investigate the impact of reinforcement and abdominal drains on the outcome of laparoscopic sleeve gastrectomy (LSG).

Methods

The present study was a prospective study that included obese patients scheduled to undergo LSG. Patients were assigned to receive drain, reinforcement, or both according to the surgeon's preference and followed up for one month after surgery. The present study's primary outcome was the identification of the association between intraoperative drain/reinforcement and the incidence of postoperative complications.

Results

A total of 125 (20.3%) patients received intraoperative drains. The proportion of postoperative morbidity was comparable between the drain and non-drain groups (3.2% versus 1.6%; p = 0.25). Patients in the drain group had similar incidence of blood transfusion (2.4% versus 1.7% in non-drain group; p = 0.43) and postoperative leakage (0.8% versus 0.2% in non-drain group; p = 0.36). The incidences of blood transfusion (p = 0.56) and reoperation (p = 0.98) were comparable between the drain and non-drain groups. There were no statistically significant differences between the drain and non-drain groups regarding postoperative mortality and wound infection (p > 0.05). On the other hand, 440 (71.3%) patients received reinforcement. The proportion of postoperative morbidity was comparable between the reinforcement and non-reinforcement groups (1.6% versus 2.8%, p = 0.07). Patients in the reinforcement group were less likely to develop postoperative bleeding (0.7% versus 4% in the non-reinforcement group; p = 0.004), while no significant difference was detected in terms of postoperative leakage (p = 0.33) and in-hospital mortality.

Conclusion

In conclusion, abdominal drainage did not reduce the complications of LSG patients. Reinforcement has some role in controlling the bleeding but not leaks. Both techniques did not significantly impact the mortality rate. In the future, additional, large randomized trials are needed to examine the gastrointestinal-related quality of life.

## Introduction

Laparoscopic sleeve gastrectomy (LSG) is receiving growing approval from patients and bariatric surgeons because of excessive weight loss and the resolution of comorbidities [1]. Unlike gastric banding and gastric bypass, the main advantages of LSG are the absence of foreign body insertion (bands), conversion to other surgical approaches, and malabsorption. However, leakage of the staple line at the proximal aspect and bleeding are the most life-threatening postoperative complications of LSG. It is known that the leading cause of gastric leakage is mechanical, for example, direct tissue injury or stapler misfiring, which leads to suture defects; gastric leakage appears within one or two days postoperatively. Moreover, the high internal pressure below the gastroesophageal junction can cause gastric leakage [2,3]. Out of 24 studies, it was reported that the mean incidence of staple-line leakage was 2.7%.

Furthermore, sepsis and death rates after unrecognized leakage exceed 50% [4]. Therefore, surgeons tend to reinforce the staple line based on one of these two approaches: oversewing the staple line or buttressing materials. Additionally, few surgeons add staple-line inversion to the procedure [5,6]. A randomized study showed that despite the longer operative time, reinforcement increased the percentage of losing weight (p < 0.01) and decreased the frequency of postoperative bleeding (p < 0.05). On the flip side, reinforcement was incorporated with an elevated incidence of vomiting and wound infection [7].

Intraoperatively, bleeding can be detected visually; however, drains can help discover bleeding postoperatively [8,9]. It was reported that during 2015-2017, the overall rate of drain insertion among surgeons was 25%. However, the American College of Surgeons (ACS) suggested that drains should not be used due to the increased risk of all-cause mortality, leak, and reoperation [10]. There is insufficient evidence regarding the role of drains and reinforcement against leak, bleeding, abscess, and death [11]. Accordingly, the current study sought to look at the impact of reinforcement or abdominal drains on the outcome of LSG. 

## Materials and methods

The study’s protocol was approved by the Institutional Review Board (IRB) of KasrAlAiny Teaching Hospital.

Study design and patients

The present study was a prospective study that included all obese patients scheduled to undergo LSG through the period from April 2019 to April 2020. Patients with hepatic disorders and/or endocrinal causes of obesity were excluded. Eligible patients were grouped according to the presence of intraoperative drain and reinforcement. The decision of intraoperative drain and reinforcement was based on the surgeon's preference. Our surgeons came from two different schools of thought. Some surgeons adopted drain insertion, staple-line reinforcement, or both in all cases they operated on irrespective of whether there were operative difficulties or not. Other surgeons did not adopt this strategy and did not put a drain or reinforce the staple line in their cases. The adoption of these two different techniques, irrespective of the patient type and operative details, enhanced patient matching between the two groups.

Surgical techniques of intraoperative drain and reinforcement

Preoperatively, patients were prepared according to the local hospital guideline; two hours before the procedure, all patients received intravenous 3rd generation cephalosporin and subcutaneous low molecular weight heparin. A five-port access technique and double-line endo staplers were used for the gastric transaction in all patients. In patients who were elected to undergo reinforcement, intracorporeal suturing with Vicryl 2/0 was done to support the staple line; the absence of leak was confirmed by the methylene blue leak test. In patients who were elected to receive intraoperative drain, an 18 French drain was placed along the staple line and fixed by silk 1. Some patients underwent both drain insertion and reinforcement according to the intraoperative surgeon's judgment and preference. Continuous 2-0 intracorporeal Vicryl sutures were used in the reinforcement.

Data collection

The following data have been collected from eligible patients in the preoperative period and during hospital stay: demographic characteristics, comorbidities, preoperative medications, presence of intraoperative drain, presence of reinforcement, and the incidence of postoperative complications, including bleeding, leakage, Continuous 2-0 intracorporeal Vicryl sutures were used in the reinforcement, wound infection, blood transfusion, reoperation, readmission, and in-hospital mortality. Patients were followed up for one month after surgery.

In our study, bleeding was defined as a hemoglobin drop by 3 g or more from the preoperative value. Moreover, bleeding was considered in patients with drain containing 100 cc or more of frank blood. Bleeding was suspected clinically by derangement of vital signs, for example, tachycardia and tachypnoea. The presence of pallor and decreased urine output were also used as clinical indicators of bleeding.

If the bleeding was suspected, close monitoring of vital signs and serial hemoglobin and hematocrit values were measured. CT scan was used in some patients to confirm the presence of hematoma in the peri-gastric region. Conservative management of bleeding was successful in most cases. The conservative strategy consisted of blood and blood products transfusion with close follow-up clinically and repeated hemoglobin measurements. Few patients did not respond to this management and reoperation was done to achieve hemostasis.

On clinical grounds, postoperative leakage was suspected if any patient developed tachycardia, tachypnoea, or fever with or without abdominal signs of peritonitis. Blood tests including inflammatory markers were done and a CT scan was used as an adjunct tool to support the diagnosis. Laparoscopic exploration was done in equivocal cases. Management of leak was mainly by re-laparoscopy, lavage, and drainage. Endoscopic stents were used as well to ensure proper control of leakage.

Outcome measures

In the current study, the primary outcome was the association between intraoperative drain/reinforcement with the incidence of postoperative complications. The secondary outcomes included predictors of postoperative bleeding and leakage among the included patients.

Statistical methodology

Data analysis was conducted using Statistical Package for the Social Sciences (SPSS), version 22.0 for Windows (IBM Corp., Armonk, NY). Quantitative variables were summarized using mean and standard deviation (SD). Qualitative variables were expressed in numbers and percentages. Independent sample t- and chi-squared tests were applied to test the hypotheses in continuous variables and categorical variables, respectively. The Pearson correlation coefficient test was performed to estimate the association between adipokines at baseline and initial BMI. The null hypothesis was rejected when the p-value was less than 0.05.

## Results

Data of 614 patients were retrieved. The mean age of the included patients was 26 ± 9 years old, and the mean BMI was 43 ± 5 kg/m^2^. Male patients represented 24.1%, and only 3.4% had diabetes mellitus. All patients received antibiotics and anticoagulants for one week after the operation. Overall, 10 patients (1.6%) had postoperative bleeding, and two patients (0.3%) developed leakage. Table 1 shows the clinical and postoperative characteristics of the included patients.

**Table 1:** The difference between the abdominal drain and no drain groups in terms of preoperative, operative, and postoperative characteristics

Variable		Drain (No. = 125)	Percentage	No Drain (No. =489)	Percentage	p-value
Pre-operative	Male	27	21.6%	122	24.9%	0.43
	Age (mean, SD)	29.02	12.95%	25.60	6.90%	0.005
	BMI (mean, SD)	44.18	8.73%	42.66	3.33%	0.059
	Diabetic	11	8.8%	32	6.5%	0.37
	HTN	15	12%	37	7.6%	0.11
	Sleep apnea	4	3.2%	8	1.6%	0.25
	Anticoagulation	13	10.4%	20	4.1%	0.013
Operative	Operative Time (mean, SD)	83.36	10.08%	82.12	10.04%	0.219
Post-operative	Morbidity	4	3.2%	8	1.6%	0.25
	Bleeding	3	2.4%	7	1.4%	0.43
	Leakage	1	0.8%	1	0.2%	0.36
	Blood transfusion	3	2.4%	8	1.6%	0.56
	Readmission	2	1.6%	1	0.2%	0.107
	Reoperation	1	2.4%	4	0.2%	0.98
	Mortality	1	0.8%	0	0%	0.203
	Wound Infection	13	10.4%	29	5.6%	0.109

A total of 125 (20.3%) patients received intraoperative drains. Patients who received drains had a significantly lower frequency of anticoagulants than patients who did not receive drains (p = 0.013). On the contrary, both groups were comparable regarding pre and intraoperative parameters (p > 0.05). The proportion of postoperative morbidity was comparable between the drain and non-drain groups (3.2% versus 1.6%; p = 0.25). Patients in drain group had similar incidence of blood transfusion (2.4% versus 1.7% in non-drain group; p = 0.43) and postoperative leakage (0.8% versus 0.2% in non-drain group; p = 0.36). The incidences of blood transfusion (p =0.56) and reoperation (p = 0.98) were comparable between the drain and non-drain groups. There were no statistically significant differences between the drain and non-drain groups regarding postoperative mortality and wound infection (p > 0.05) (Table 2).

**Table 2:** The difference between the abdominal reinforcement and no reinforcement groups in terms of preoperative, operative, and postoperative characteristics

Variable		Reinforcement group (No. =440)	Percentage	No Reinforcement (No. =174)	Percentage	p-value
Pre-operative	Male	108	24.5%	41	23.2%	0.717
	Age (mean, SD)	28.00	9.01%	22.17	5.78%	<0.001
	BMI (mean, SD)	43.06	5.44%	42.81	3.72%	0.520
	Diabetic	18	4.1%	3	1.77%	0.138
	HTN	13	3%	4	2.3%	0.789
	Sleep apnea	3	0.7%	2	1.1%	0.628
	Anticoagulation	11	2.5%	2	1.1%	0.367
Operative	Operative Time (mean, SD)	85.20	8.99%	75.25	8.99%	<0.001
Post-operative	Morbidity	7	1.6%	5	4%	0.07
	Bleeding	3	0.7%	7	4%	0.004
	Leakage	0	0%	2	0.5%	0.33
	Blood transfusion	5	1.1%	7	4%	0.017
	Readmission	3	0.7%	0	0%	0.561
	Reoperation	2	0.5%	2	1.1%	0.325
	Mortality	1	0.2%	0	0%	NA
	Wound Infection	27	6.1%	15	8.6%	0.27

**Figure 1:**
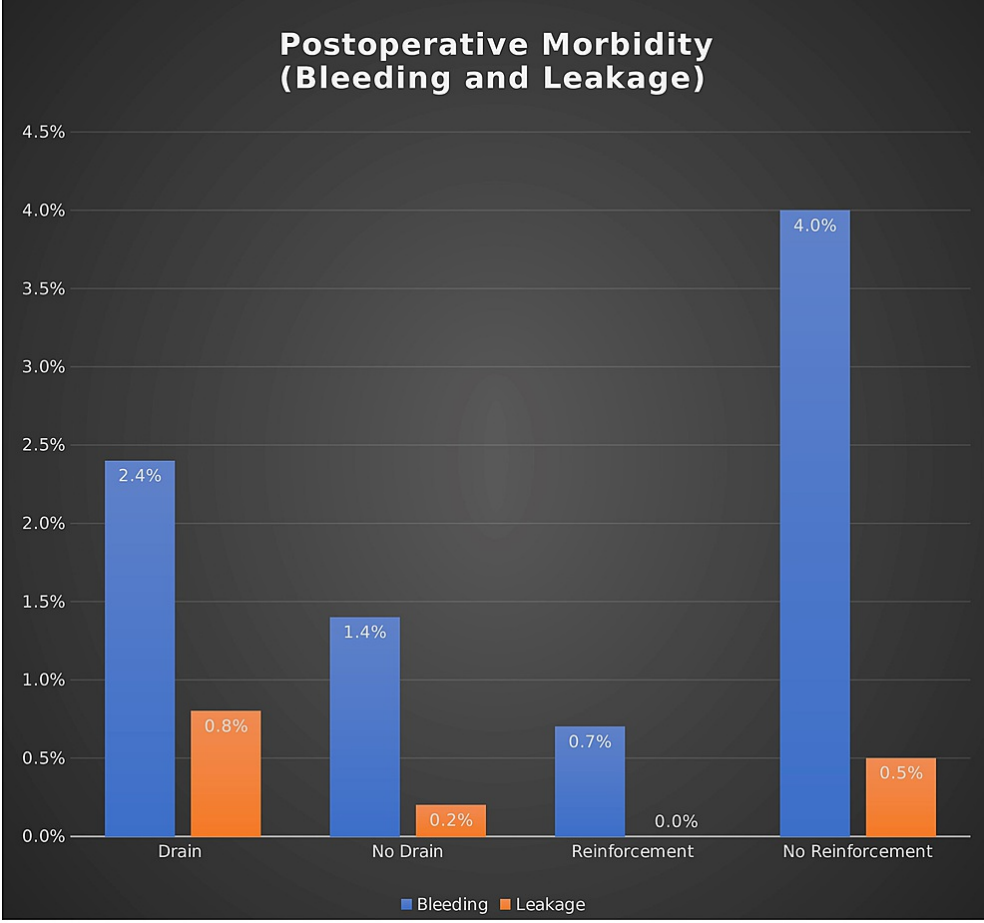
The difference in postoperative morbidity rate (bleeding and leak)

On the flip side, 440 (71.3%) patients received reinforcement. The proportion of postoperative morbidity was comparable between the reinforcement and non-reinforcement groups (1.6% versus 2.8%, p = 0.07). Patients in the reinforcement group were less likely to develop postoperative bleeding (0.7% versus 4% in the non-reinforcement group; p = 0.004), while no significant difference was detected in terms of postoperative leakage (p = 0.33) and in-hospital mortality (Figure 1).

## Discussion

In the current study, our findings showed that abdominal drainage during bariatric surgery was associated with a similar incidence of postoperative morbidity, bleeding, leakage, blood transfusion, and reoperation to that of patients who did not receive drains. These findings contradict the recommendations of the ACS, who reported that drain was associated with 30% higher odds of postoperative leakage, in addition to high rates of reoperation, readmission, and morbidities [10]. Such heterogeneity stems from the fact that, in our study, abdominal drainage was placed according to the surgeons’ preference rather than the preoperative or intraoperative condition of the patient. Some surgeons prefer to place drains for up to 72 hours to remove the collected fluids and detect intra-abdominal bleeding due to staple-line leaks, which may associate with upper gastrointestinal bleeding [12,13]. Thus, the patient may present with hematemesis or melena. Another origin of bleeding may be the abdominal wall's injury or liver during the trocar entry [14]. Early detection of gastric leakage can reduce the risk of mortality.

Nevertheless, it may become challenging to identify postoperative leaks since obese patients are not clinically reliable [15,16]. Female diabetic patients with higher BMI and patients with sleep disorders are more likely to have a drain placed [17]. Regarding the type of surgery, the lowest rate of drain was found in LSG (16.7%), followed by laparoscopic Roux-en-Y gastric bypass (LRYGB) (29%) and revisions (30%) [10].

Dourmouras and his colleagues thought that drain should not be used anymore, as it increases the risk of all-cause mortality [18]. Curro et al. compared 100 LSG procedures with drains and 100 without drains. They reported no significant difference between both groups. Moreover, they showed that drains have no protection role against abscess formation and with no role in fistula detection [19]. Similarly, Albanopoulos et al. [9] demonstrated that reoperation rates were not reduced with drains. They recommended a routine physical examination, white blood cell (WBC) count, complete blood count (CBC), and C-reactive protein (CRP) measurements to control these patients. The current evidence suggests that drains do not show clear benefits; they only can be used to determine leakage and bleeding. Besides, drains cause severe discomfort associated with nausea, vomiting, and abdominal pain.

Regarding reinforcement, it is available with various cartridge sizes to provide sufficient tissue apposition for different thicknesses and strengths without causing ischemia or tissue destruction [20,21]. Time is considered a vital factor in stapling mechanics. Suitable time for tissue compression and tissue creep allows for adequate tensile strength and preservation of tissue. Absorbable and nonabsorbable buttressing materials are widely available in the setting of bariatric surgery [22,23]. In an animal model, the staple-line enforcement showed significant efficacy with higher intraluminal pressures using bovine pericardium when compared with non-buttressing. Besides, the use of buttressing materials significantly reduces intraoperative bleeding and, therefore, operation time [24].

In this study, we found that the incidence of postoperative morbidity and wound infection of the reinforcement group was higher when compared with the non-reinforcement group. On the other hand, our findings showed that patients in the reinforcement group were less likely to develop postoperative bleeding (p = 0.004). Similarly, it was reported that the effect of reinforcement materials on bleeding was better than leakage [25]. In particular circumstances such as obstruction, these materials have higher burst pressure that could be useful. The longstanding safety of nonabsorbable materials has also been debatable. Bovine pericardial strips are often described by the author as foreign bodies, and reports of staple relocation or migration to the stomach and GI tract are available [26]. However, some used nonabsorbable material, and they did not discuss these complications [27]. A large systematic review concluded that reinforcement has no clear benefit [25].

This study's main limitation is the absence of randomization between groups, as the allocation was based on surgeon judgment and preference since we do not have clear or solid guidelines regarding this topic. Moreover, we did not investigate the quality of life related to gastrointestinal outcomes in both groups. Some parameters, such as excess losing weight, hospital stay, and intraoperative blood loss time were not assessed.

## Conclusions

In conclusion, abdominal drainage did not reduce the complications of LSG patients. Reinforcement has some role in controlling bleeding but not leaks. However, both techniques did not significantly impact the mortality rate. Furthermore, large randomized trials are needed to examine the gastrointestinal-related quality of life.
